# Two Cases of Primary Cold Agglutinin Disease Associated with Megaloblastic Anemia

**DOI:** 10.1155/2015/913795

**Published:** 2015-03-30

**Authors:** Shinsaku Imashuku, Naoko Kudo, Katsushige Takagishi, Katsuyasu Saigo

**Affiliations:** ^1^Department of Laboratory Medicine, Uji-Tokushukai Medical Center, Uji 611-0042, Japan; ^2^Division of Hematology, Takasagoseibu Hospital, Takasago 676-0812, Japan; ^3^Department of Internal Medicine, Uji-Tokushukai Medical Center, Uji 611-0042, Japan

## Abstract

We report two cases of primary cold agglutinin disease (CAD) associated with megaloblastic anemia in Japanese elderly patients. Case 1 was a 67-year-old male and Case 2 was a 55-year-old male. Both patients were diagnosed with primary CAD, with continuously high cold agglutinin titers (1 : >8,192 and 1 : 16,834, resp.), monoclonal IgM-kappa light chains, and no underlying disease. In addition, both patients had megaloblastic anemia due to vitamin B12 deficiency. One patient received rituximab and both received vitamin 12 supplementation. To date, no cooccurrence of primary CAD and megaloblastic anemia has been emphasized. Thus, the association of these hematological diseases may be incidental; however, given that CAD is an autoimmune disease which may show antibodies against intrinsic factor and gastric parietal cells, this association was thought to be probably not a coincidence. Clinicians should be aware of the possible simultaneous presence of autoimmune hemolytic/megaloblastic anemia in patients with primary CAD.

## 1. Introduction

Cold agglutinin disease (CAD), or cold antibody autoimmune hemolytic anemia, is characterized by mild anemia with reticulocytosis, positive direct Coombs test, elevated levels of lactate dehydrogenase (LDH), low levels of serum haptoglobin, and high titers of cold agglutinin [1–3]. CAD manifests as either a primary disease, that is, chronic CAD, or secondary to Waldenström's macroglobulinemia (WM) or B-cell type malignant lymphoma [4, 5]. Secondary CAD also occurs in association with systemic lupus erythematosus [6] or transiently upon Epstein-Barr virus or mycoplasma pneumoniae infection [7]. Cold agglutinins, which are specific for the I-antigen expressed on the surface of red blood cells, belong to the IgM subclass and, in the majority of patients with primary CAD, are monoclonal IgM-kappa antibodies [1–3]. Primary CAD is most often seen in elderly patients (median age at onset is 67 years (range 30–92 years)) and the incidence rate is 1 per 1 million people per year [2]. Primary CAD may develop in association with various hematological/immunological diseases, including pernicious anemia [8] and common variable immunodeficiency (CVID) [9]. Here, we report the cases of two elderly Japanese patients with primary CAD who showed clinical features of megaloblastic anemia due to decreased vitamin 12 levels. In addition, one of these patients also showed probable CVID in addition to typical CAD symptoms.

## 2. Case Presentation

### 2.1. Case  1

**Figure 1:**
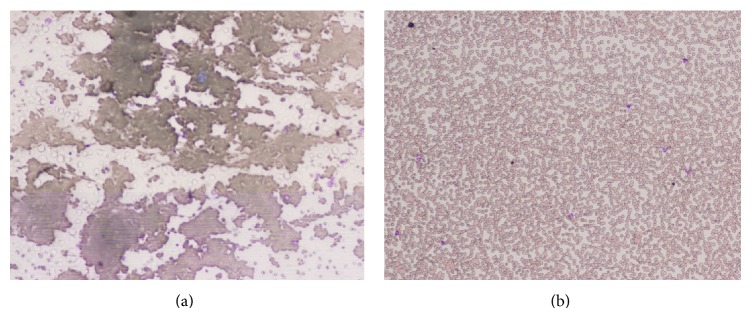
Peripheral blood smear showing (a) red blood cell agglutination at room temperature and (b) no agglutination after warming at 37°C (Wright-Giemsa stain; original magnification ×100).

A 67-year-old male was diagnosed with CAD in 2009. Since then, over the past 3 years, he had maintained Hb levels at 15.0 to 16.5 g/dL but complained of peripheral coldness and cyanosis of the limbs in association with Raynaud's phenomenon, particularly in cold seasons; however, he did not receive any specific therapy. The patient was hospitalized due to progression of anemia and hemoglobinuria in December 2012. In the summer of that year he had Hb level at 16.2 g/dL and then became anemic over the fall-to-winter period. His prior medical history revealed alcoholic liver dysfunction, mild diabetes mellitus, and hypertension. There was no history of inappropriate dietary intake or drug use and no recent ongoing excess alcohol use. On admission, the patient (height 167 cm and body weight 73.4 kg) was anemic (Hb 8.1 g/dL) and slightly icteric, with total bilirubin levels of 2.5 mg/dL. He also had macrocytic anemia. A peripheral blood film revealed marked red blood cell agglutination (Figure 1). A CT scan showed no lymph adenopathy or splenomegaly. The laboratory data are summarized in Table 1. During the 3 years prior to hospitalization, his cold agglutinin titer remained high (1 : 2,048); however, upon hospitalization it was 1 : >8,192. He also had monoclonal M-proteins (IgM-kappa) but normal IgG, IgA, and IgM; however, complement levels were low (Table 1). In this case, no bone marrow analyses were performed; however, during the entire course of CAD, he did not show any signs of lymphoproliferative diseases (serum sIL-2R remained within normal range and there were negative CT findings). In addition, the patient had low vitamin 12 levels, confirming megaloblastic anemia, with positive anti-intrinsic factor as well as antiparietal cell antibodies. Gastrointestinal endoscopy revealed atrophic gastritis. In addition to vitamin B12 supplementation (mecobalamin 500 *μ*g × 3/day), he was treated with four doses of weekly rituximab (375 mg/m^2^/dose), which increased the Hb levels from 8.1 g/dL to 14.7 g/dL and reduced serum LDH levels from 1,119 IU/L to 201 IU/L 2 months later. MCV was normalized in 2 months following vitamin B12 administration. For the last 2 years, he has been doing well without rituximab maintenance therapy, with Hb levels >15.0 g/dL, LDH levels around 160 IU/L, a cold agglutinin titer of 1 : 2,048, and no episodes of acute hemolysis.

### 2.2. Case  2

**Figure 2:**
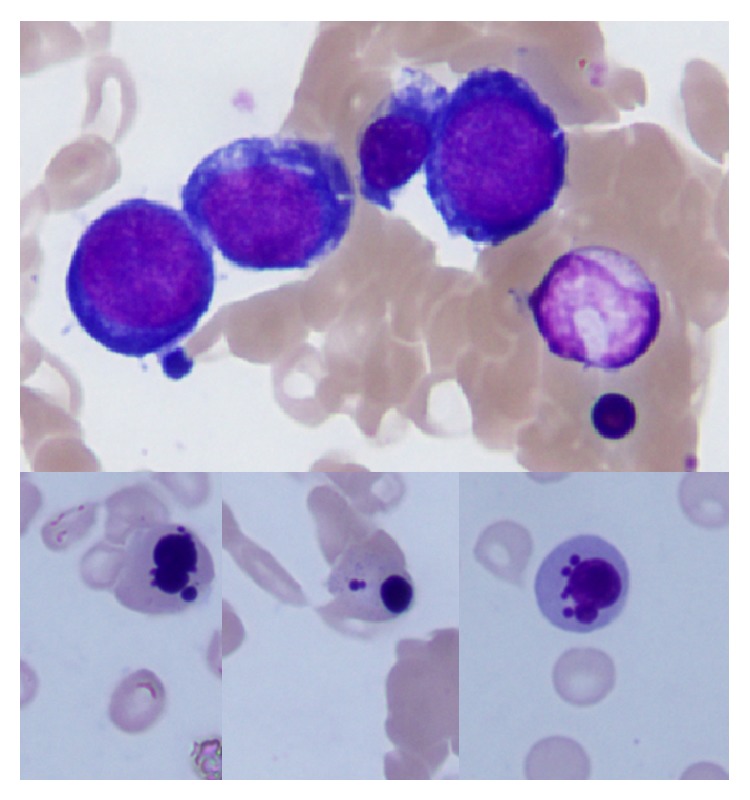
Bone marrow smear from Case 2 showing megaloblastic changes associated with the dyserythropoietic features of polychromatic erythroblasts.

A 55-year-old male (height 170 cm and body weight 65.4 kg) was diagnosed with CAD in 2012 when he did not show anemia, apart from a slight increase in total bilirubin levels (2.2–3.0 mg/dL). One year later, after exposure to cold, he again visited a clinic and was found to be mildly anemic (Hb 8.0 g/dL); however, he was not systematically tested or treated. In his history he had inappropriate dietary intake but no drug use. In February 2014, he was hospitalized via emergency medical transport due to the fact that he was severely anemic (Hb 4.3 g/dL) as well as icteric, with a peripheral blood smear showing significant red blood cell agglutination (data not shown). A CT scan revealed no lymphadenopathy, but pulmonary edema, bilateral pleural fluid, and mild splenomegaly were noted. The laboratory data are summarized in Table 1. His cold agglutinin level was extremely high (1 : 16,834) and he had monoclonal M-proteins (IgM-kappa) in association with hypogammaglobulinemia (IgG, 584 mg/dL) and hypocomplementemia. In addition, he had megaloblastic anemia associated with low serum levels of vitamin B12 and folate. A bone marrow smear showed an increase in megaloblasts associated with dyserythropoietic features of polychromatic erythroblasts (Figure 2), with an M/E ratio of 0.92; these data were compatible with a diagnosis of both hemolytic and megaloblastic anemia. No increase in the number of myeloma cells or lymphoma cells was noted. Bone marrow cells showed a normal karyotype (46 XY; 20/20). He was treated with a heated RBC transfusion (a total of 10 U) and received oral folate (5 mg/day × 3/day) and vitamin 12 (mecobalamin 500 *μ*g × 3/day) but declined intravenous rituximab therapy. Unfortunately, this patient was discharged not for medical recovery but for his personal reasons and became lost to follow-up.

## 3. Discussion

**Figure 3:**
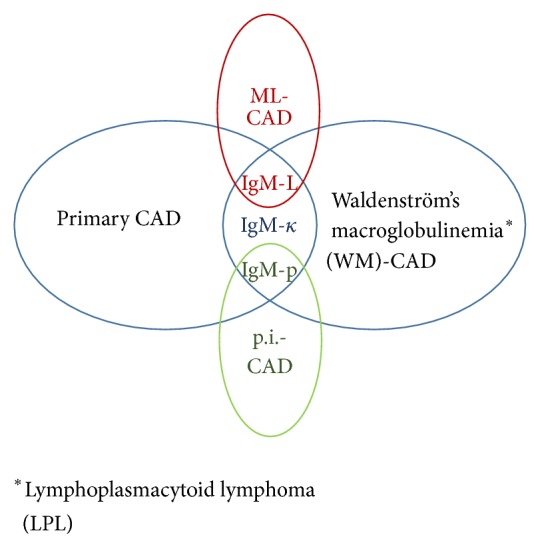
The majority of primary CAD and WM-associated CAD cases (>70%) show cold agglutinins of monoclonal IgM-kappa antibodies. On the other hand, lymphoma-associated CAD is associated with monoclonal IgM-lambda antibodies [2]. Cold agglutinins associated with postinfectious diseases such as Epstein-Barr virus or mycoplasma pneumoniae infection-related CAD can be polyclonal [1]. CAD: cold agglutinin disease; ML: malignant lymphoma; WM: Waldenström's macroglobulinemia; p.i.: postinfection; *κ*: kappa; L: lambda; p: polyclonal.

Both patients showed clinical features typical of chronic CAD with high titers of cold agglutinin. Direct Coombs test (DCT) was negative, as shown in Table 1, for both patients with use of wide spectrum antiserum. It was reasoned that our patients were not in a hemolytic phase when DCT was tested. In fact, in patients with mycoplasma pneumoniae-related CAD, the incidence of patients with a positive DCT was shown to fall with time [10]. After the interval of 2-3 years from the first recognition of CAD symptoms, both of our patients required intensive therapy for hemolytic anemia upon hospitalization. CAD may develop in association with WM, B-cell type lymphoma, SLE, or upon Epstein-Barr virus or mycoplasma pneumoniae infection [4–7]; however, these two patients had no such underlying diseases. In addition, both patients showed monoclonal IgM-kappa antibodies, compatible with primary CAD or WM, differentiated from other types of CAD (Figure 3). However, WM was ruled out; thus primary CAD was diagnosed. In addition to clinical symptoms and laboratory data typical of CAD, both patients had megaloblastic anemia due to vitamin B12 deficiency. Macrocytic anemia in our cases was not false elevations of the MCV due to cold agglutinins [11]. The reduced complement levels (C3, C4, and CH50 were all reduced) observed in these patients were similar to those reported in previous studies [12, 13]. Ulvestad et al. reported that, of 15 primary CAD patients, nine showed decreased serum C3, 11 showed C4, and six showed reduced CH50, indicating that patients with CAD experience continuous complement consumption [13]. Furthermore, Case 2 had mild hypogammaglobulinemia.

In primary CAD, both the cold-reactive antibodies and the IgG-kappa antibodies on circulating red cells play a role in the various hematological/immunological abnormalities [12]. However, few studies have reported cooccurrence of CAD and megaloblastic anemia. Such an association in our cases might be incidental; however, given that CAD is an autoimmune disease caused by multiple autoantibodies, it may not be a coincidence, particularly as one study reported a case of CAD with pernicious anemia [8]. It is possible that the megaloblastic anemia in our cases was actually pernicious anemia, a complex disorder consisting of hematological, gastric, and immunological alterations, as we confirmed the evidence for antibodies against intrinsic factor or gastric parietal cells in Case  1, although antibodies against the intrinsic factor are not specific pernicious anemia. Other factors such as chronic use of alcohol in Case 1 and inappropriate dietary take in Case 2 might also have played an additional role for vitamin B12 deficiency and macrocytosis.

Primary CAD could also be known to be associated with other immunological aberrations. Previous studies report cases of CAD with agammaglobulinemia or CVID [9, 14, 15]. Indeed, autoimmune manifestations are associated with CVID in about 20% to 25% of cases [16]. Case 2 may have had CVID, which may show a link with the CAD development. In terms of treatment, conventional therapies for CAD are not very effective. However, a recent study showed that rituximab is effective [17]. Case 1 achieved remission lasting more than 2 years after receiving four doses of rituximab alone; however, Case 2 declined rituximab and his disease remains active. A combination of rituximab plus alkylating agents, such as oral cyclophosphamide [18], fludarabine [19], or bendamustine [20], appears to be an effective treatment for refractory CAD cases. It is possible that the patients described herein may require such intensive therapeutic measures in the future.

## Figures and Tables

**Table 1:** Laboratory data of 2 CAD cases.

	Case 1	Case 2
Age (years)/sex	67/M	55/M
WBC (3000–8500)/*μ*L	9900	6300
Hb (12.5–17.5) g/dL	8.1	4.3
MCV (84.6–100.6) fL	115	110
Reticulocytes (0.3–1.1) %	11.5	8.2
PLTs (115,000–305,000)/*µ*L	198,000	147,000
Haptoglobin (19–170) mg/dL	67	2
AST (13–37) IU/L	45	25
ALT (8–45) IU/L	37	14
LDH (122–228) IU/L	1119	1021
Total bilirubin (0.3–1.3) mg/dL	2.50	7.94
Direct bilirubin (0.1–0.3) mg/dL	1.10	1.30
Total protein (6.7–8.3) g/dL	7.0	5.9
Albumin (4.1–5.2) g/dL	4.0	4.3
BUN (7.8–18.9) mg/dL	19.2	8.3
Creatinine (0.64–1.11) mg/dL	0.88	0.86
CRP (0–0.29) mg/dL	0.60	0.92
Direct Coombs test^*^	Negative	Negative
Cold agglutinin titer	1 : >8,192	1 : 16,384
M-protein	Positive (IgM-kappa)	Positive (IgM-kappa)
Cryoglobulin	Negative	NT
HBV/HCV	Negative/negative	Negative/NT
ANA	<×40	<×40
Soluble IL-2R (122–496) U/mL	437	NT
IgG/IgA/IgM (820–1740/90–400/31–200)	1065/554/217	584/NT/164
C3/C4/CH50 (80–140/11–34/30–45)	60/5.0/10.8	56/6.3/7.0
Folate (3.6–12.9) ng/mL	10.9	3.2
Vitamin B12 (233–914) pg/mL	85	173
Anti-IF-Ab	Positive	NT
Anti-PC-Ab	Positive	NT

^∗^Employed with wide spectrum antiserum, not with monospecific anti-C3 antibody, WBC: white blood cell counts, PLTs: platelet counts, AST: aspartate aminotransferase, ALT: alanine aminotransferase, LDH: lactate dehydrogenase, BUN: blood urea nitrogen, CRP: C-reactive protein, and ANA: antinuclear antibody; units for IgG, IgA, IgM, C3, and C4 are mg/dL, and unit for CH50 is U/mL; IF: intrinsic factor, PC: parietal cell, Ab: antibody, and NT: not tested.
